# Supplementary figures and images for: Microbial dark matter filling the niche in hypersaline microbial mats
Source: Microbiome. 2020 Sep 16;8:135. doi: 10.1186/s40168-020-00910-0 (PMC7495880; doi:10.1186/s40168-020-00910-0)

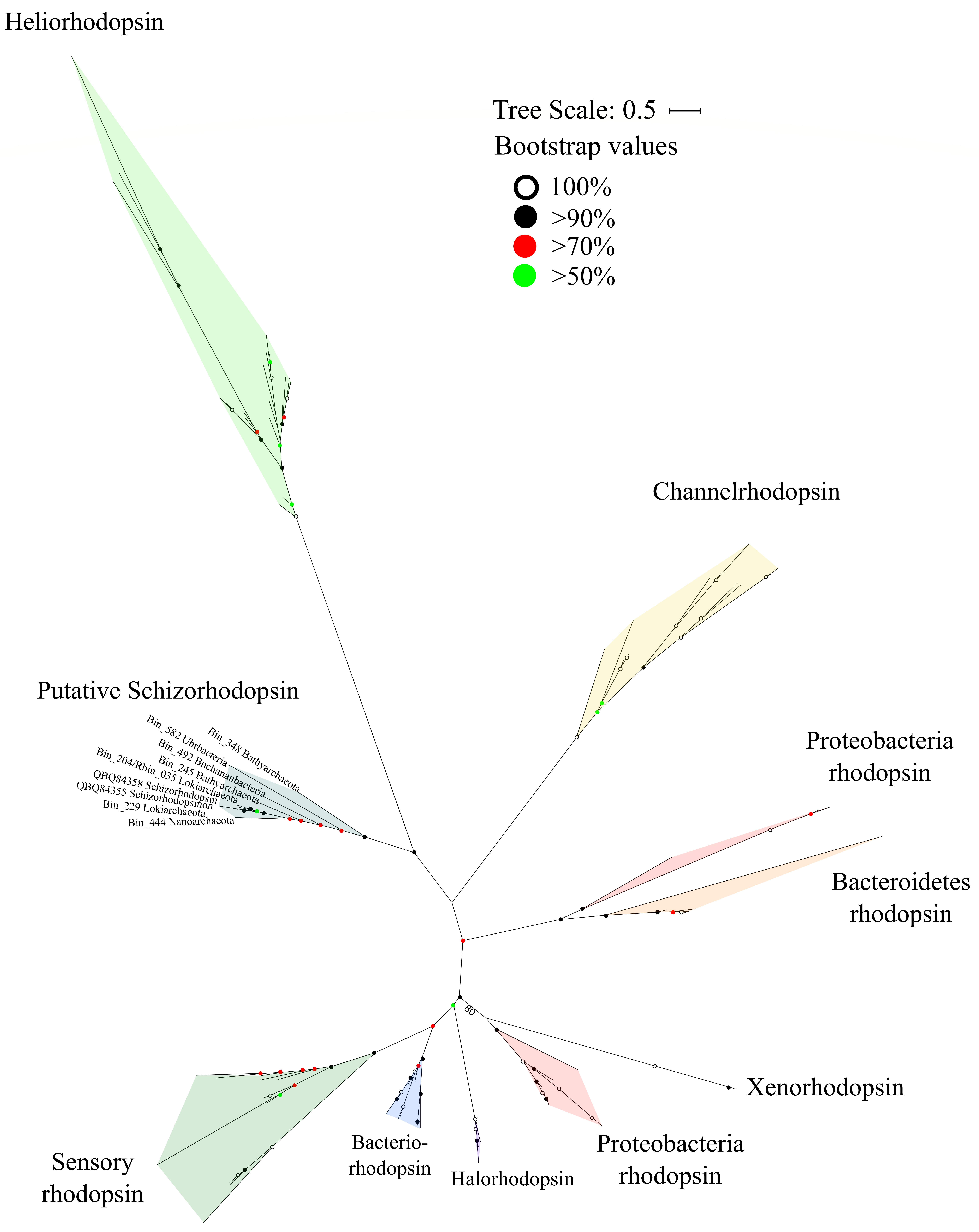

Supplement: Supplementary file 3 — Additional file 2: Figure S1. Unrooted maximum-likelihood phylogenetic tree of putative rhodopsin in Shark Bay MDM MAGs. Maximum-likelihood phylogenetic tree constructed with rhodopsin gene found in the MDM MAGs with 1000 bootstrap replications. Lokiarchaeota, Bathyarchaeota, Uhrbacteria, Buchananbacteria and an unclassified archaeon encode rhodopsin clustered in the same group with the novel, recently discovered schizorhodopsin [7]. Circular dots of different colors represent bootstrap values. Rhodopsin sequences in this study, reference sequences and BLAST results are listed in Additional file 19: Table S2. [file 40168_2020_910_MOESM2_ESM.tiff]

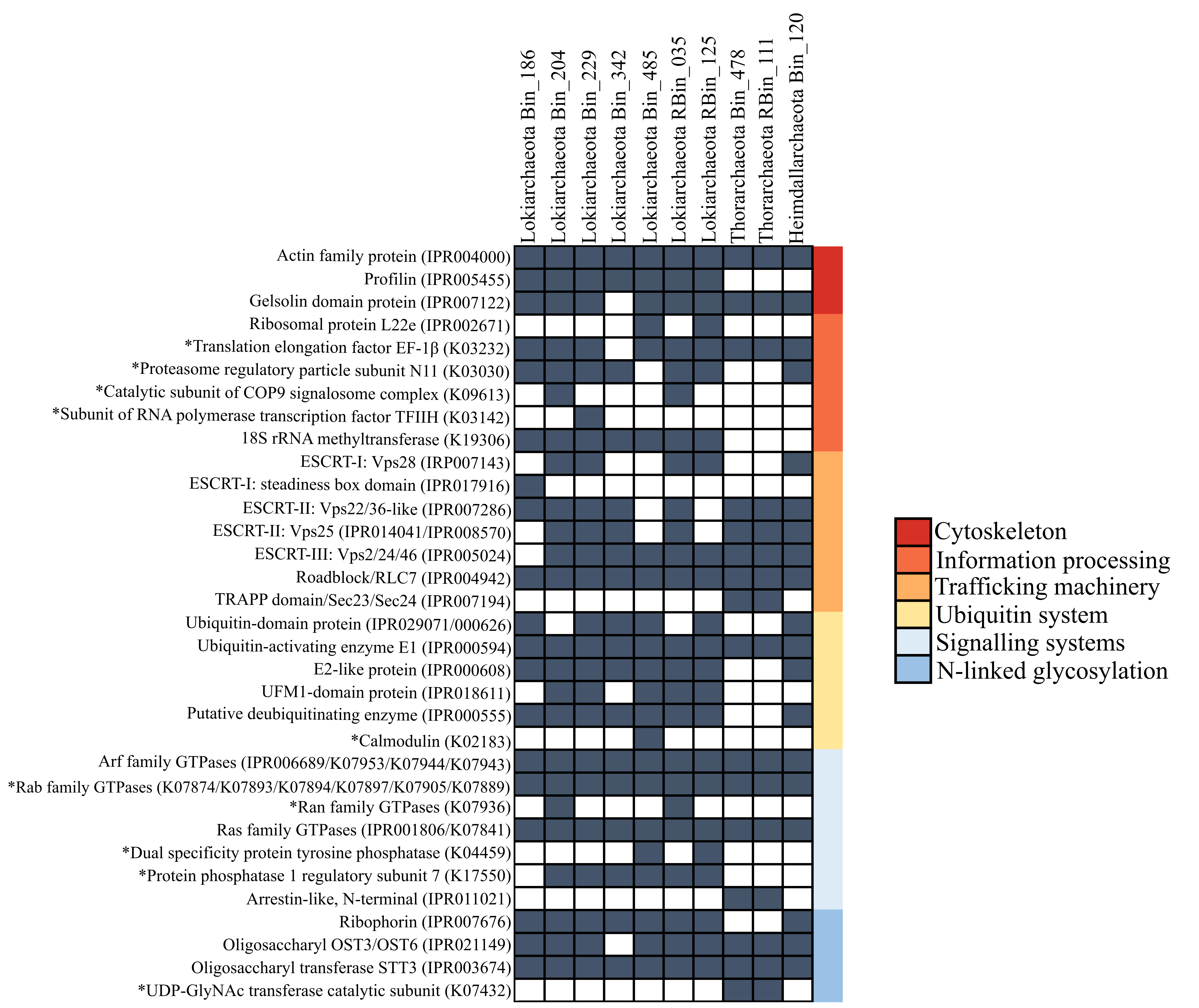

Supplement: Supplementary file 4 — Additional file 3: Figure S2. Eukaryotic Signature Proteins (ESPs) in the MAGs of Asgard archaea. MAGs were annotated using Interproscan [77] and GhostKoala [78] and confirmed using HHpred [79] and BLAST [80]. Shark Bay Asgard archaea were found to contain ESP likely involved in cytoskeleton dynamics, information processing, trafficking machinery, signalling systems as well as eukaryotic-like N-linked glycosylation. * indicates newly identified ESP. Dark and white boxes indicate presence and absence of genes respectively. [file 40168_2020_910_MOESM3_ESM.tiff]

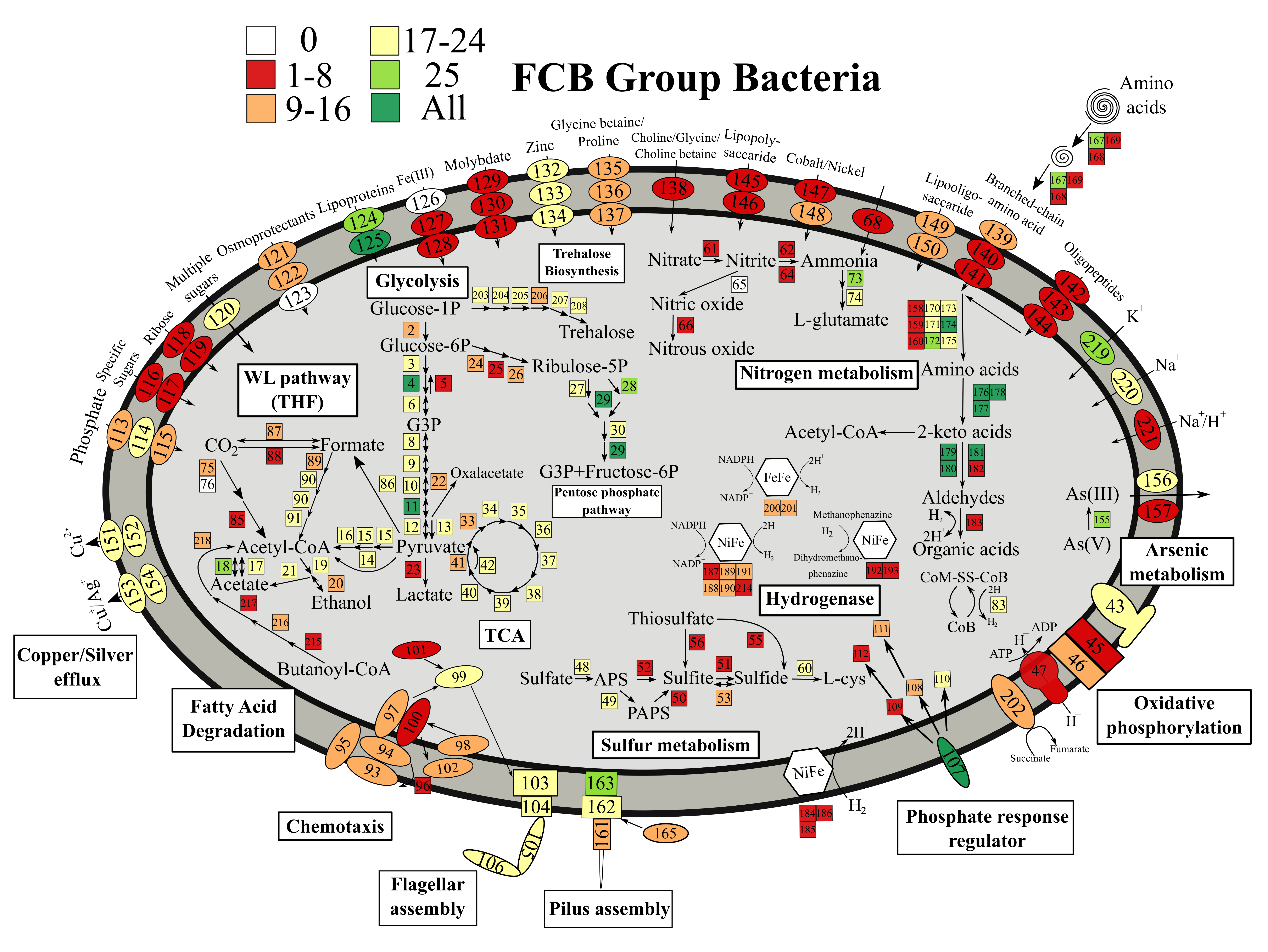

Supplement: Supplementary file 5 — Additional file 4: Figure S3. Metabolic potential of FCB (Fibrobacteres-Chlorobi-Bacteroidetes) group bacteria. A metabolic map summarising the genomic potential and metabolic capacities of the 26 MAGs affiliated with the FCB group. Numbers represent specific genes in given pathways and the corresponding genes are listed in Additional file 20: Table S3. Different colors in the square boxes represent different numbers of MAGs encoding the genes, while white square boxes indicate the absence of the genes. TCA, tricarboxylic acid cycle; THF, tetrahydrofolate; WL pathway, Wood-Ljungdahl pathway; PAPS, 3’-phosphoadenylyl sulfate; APS, Adenylyl sulfate. [file 40168_2020_910_MOESM4_ESM.tiff]

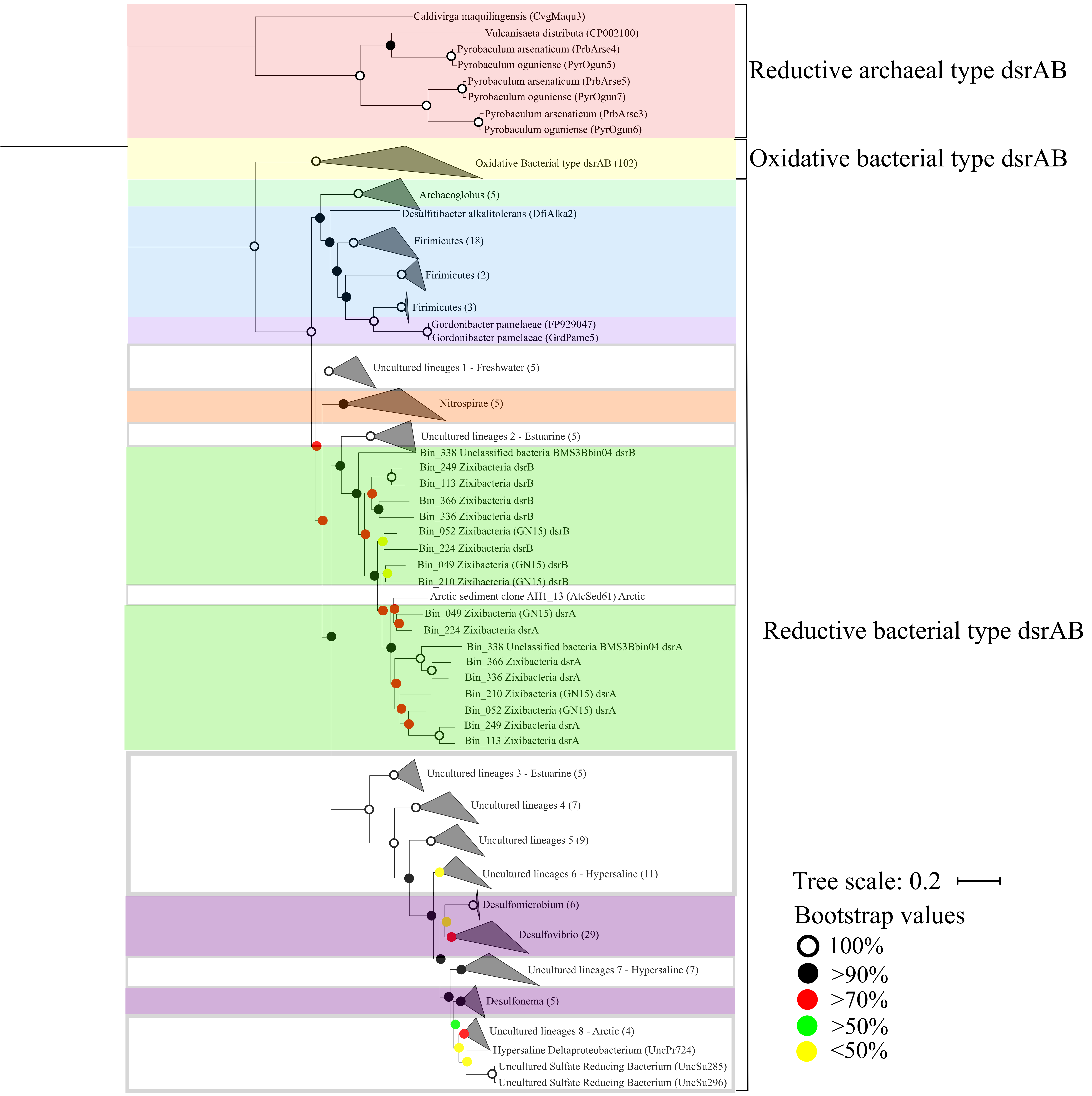

Supplement: Supplementary file 6 — Additional file 5: Figure S4. Maximum-likelihood phylogenetic tree of dsrAB in Shark Bay MDM MAGs. Maximum-likelihood phylogenetic tree was constructed with reference dsrAB sequences from the dsrAB database [105], with 1000 bootstrap replications. dsrAB genes found in the present study are classified as reductive bacterial type dsrAB and are highlighted in green. Circular dots of different colors represent bootstrap values. dsrAB sequences found in the MDM MAGs are listed in Additional file 24: Table S7. Branches shaded red indicates reductive archaeal type dsrAB, yellow shade indicates oxidative bacterial type dsrAB, light green indicates Archaeoglobus lineages, light blue indicates Firmicutes lineages, light purple indicates Actinobacteria lineages, orange represents Nitrospirae lineages, purple represents Deltaproteobacteria lineages, green represent dsrAB in the present study and no shades represent uncultured/environmental lineages. [file 40168_2020_910_MOESM5_ESM.tif]

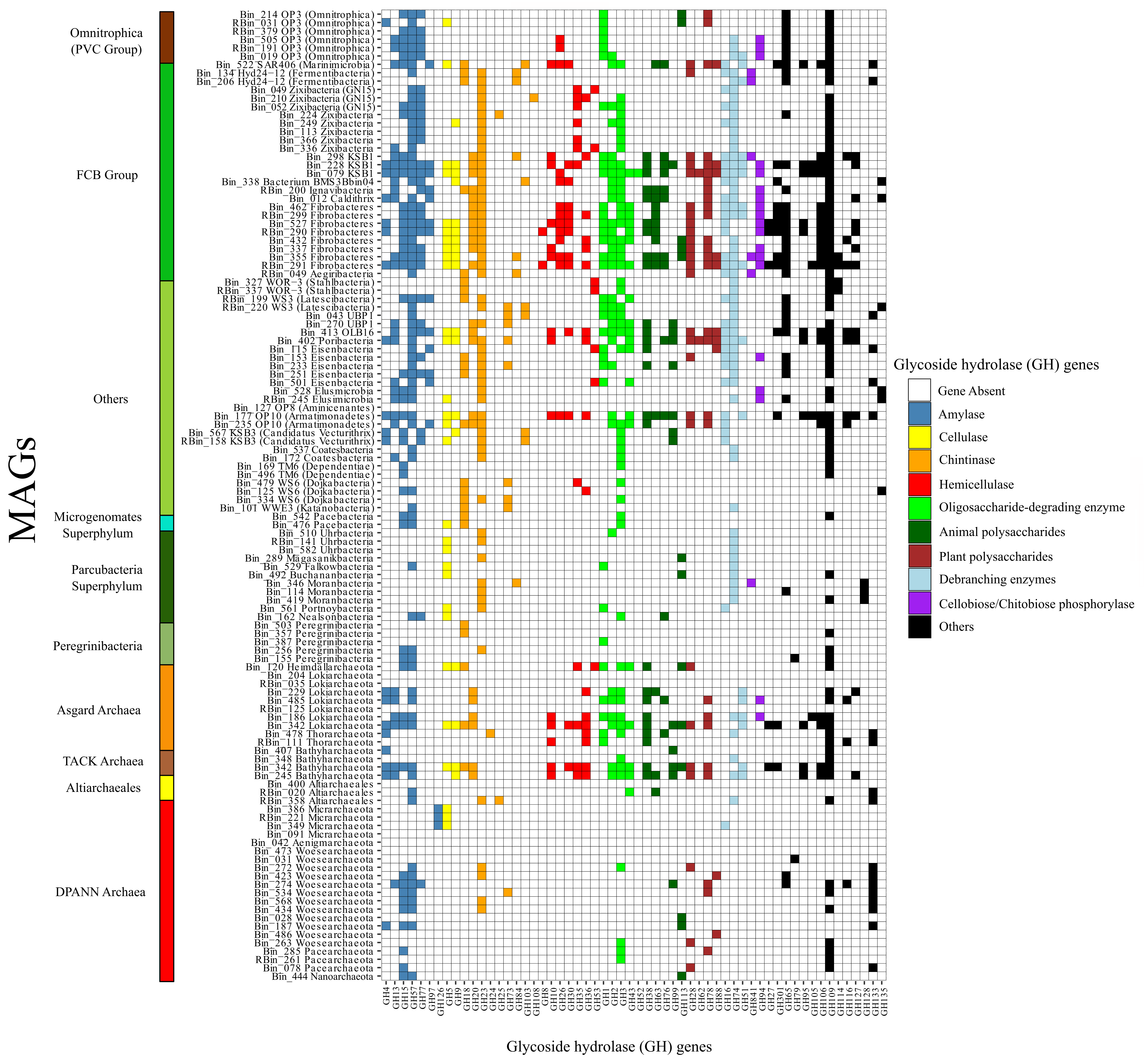

Supplement: Supplementary file 7 — Additional file 6: Figure S5. Color-coded table indicating major carbohydrate-active enzymes (CAZy) in MDM MAGs. X-axis indicates different types of glycoside hydrolase (GH) genes in the CAZy database and y-axis represent MAGs of microbial dark matter. White indicates absence of GH genes in the MAGs. Color panel on the left represents different groups of MDM MAGs according to Fig. 1. [file 40168_2020_910_MOESM6_ESM.tiff]

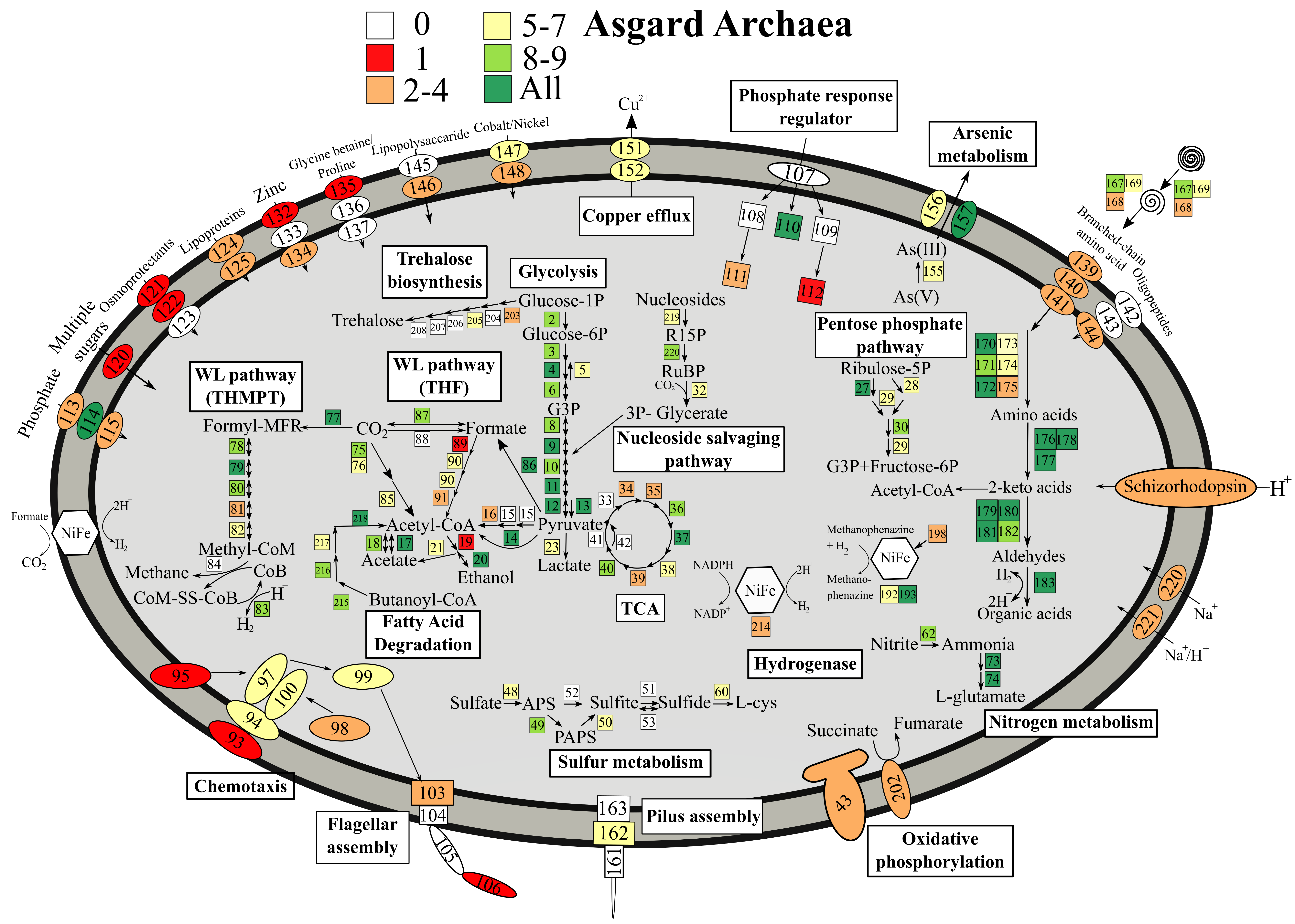

Supplement: Supplementary file 8 — Additional file 7: Figure S6. Metabolic potential of Asgard archaea. A metabolic map summarising the genomic potential and metabolic capacities of the 10 MAGs affiliated with Asgard archaea. Numbers represent specific genes in given pathways and the corresponding genes are listed in Additional file 20: Table S3. Different colors in the square boxes represent different numbers of MAGs encoding the genes, while white square boxes indicate the absence of the genes. TCA, tricarboxylic acid cycle; THF, tetrahydrofolate; THMPT, tetrahydromethanopterin; WL pathway, Wood-Ljungdahl pathway; PAPS, 3’-phosphoadenylyl sulfate; APS, Adenylyl sulfate. [file 40168_2020_910_MOESM7_ESM.tiff]

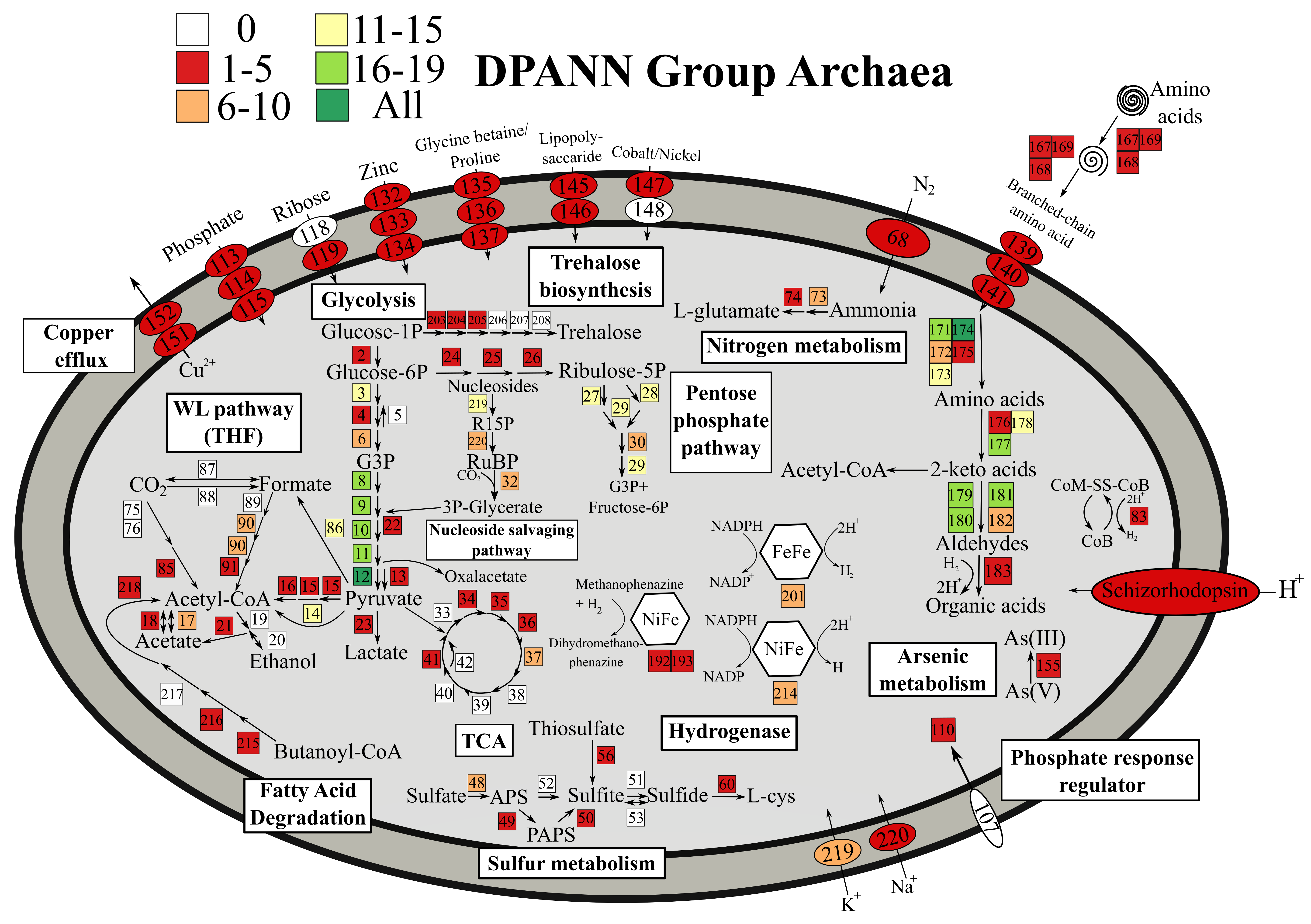

Supplement: Supplementary file 9 — Additional file 8: Figure S7. Metabolic potential of DPANN archaea. A metabolic map summarising the genomic potential and metabolic capacities of the 21 MAGs affiliated with DPANN archaea. Numbers represent specific genes in given pathways and the corresponding genes are listed in Additional file 20: Table S3. Different colors in the square boxes represent different numbers of MAGs encoding the genes, while white square boxes indicate the absence of the genes. TCA, tricarboxylic acid cycle; THF, tetrahydrofolate; THMPT, tetrahydromethanopterin; WL pathway, Wood-Ljungdahl pathway; PAPS, 3’-phosphoadenylyl sulfate; APS, Adenylyl sulfate. [file 40168_2020_910_MOESM8_ESM.tiff]

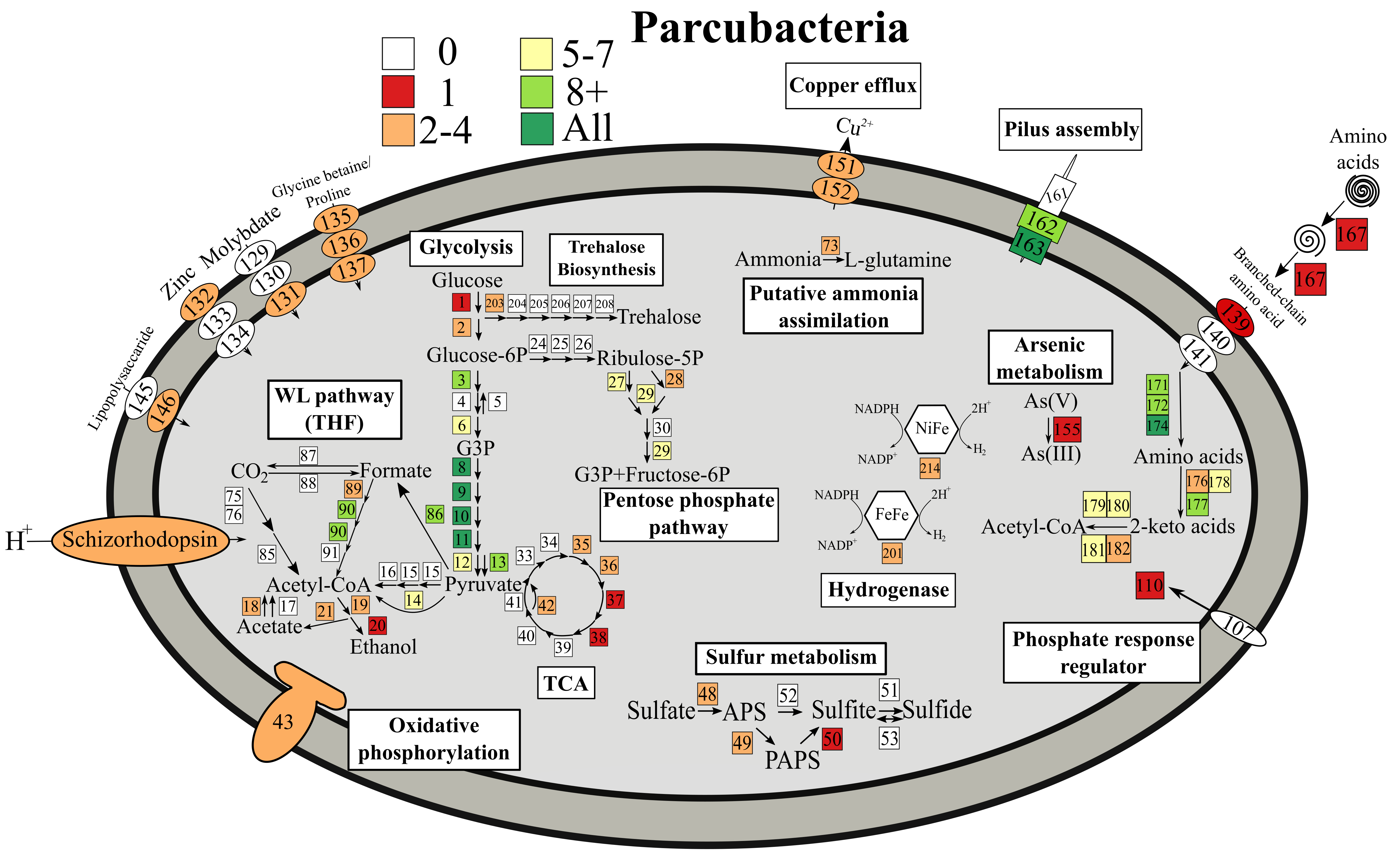

Supplement: Supplementary file 10 — Additional file 9: Figure S8. Metabolic potential of Parcubacteria. A metabolic map summarising the genomic potential and metabolic capacities of the 11 MAGs affiliated with Parcubacteria. Numbers represent specific genes in given pathways and the corresponding genes are listed in Additional file 20: Table S3. Different colors in the square boxes represent different numbers of MAGs encoding the genes, while white square boxes indicate the absence of the genes. TCA, tricarboxylic acid cycle; THF, tetrahydrofolate; THMPT, tetrahydromethanopterin; WL pathway, Wood-Ljungdahl pathway; PAPS, 3’-phosphoadenylyl sulfate; APS, Adenylyl sulfate. [file 40168_2020_910_MOESM9_ESM.tiff]

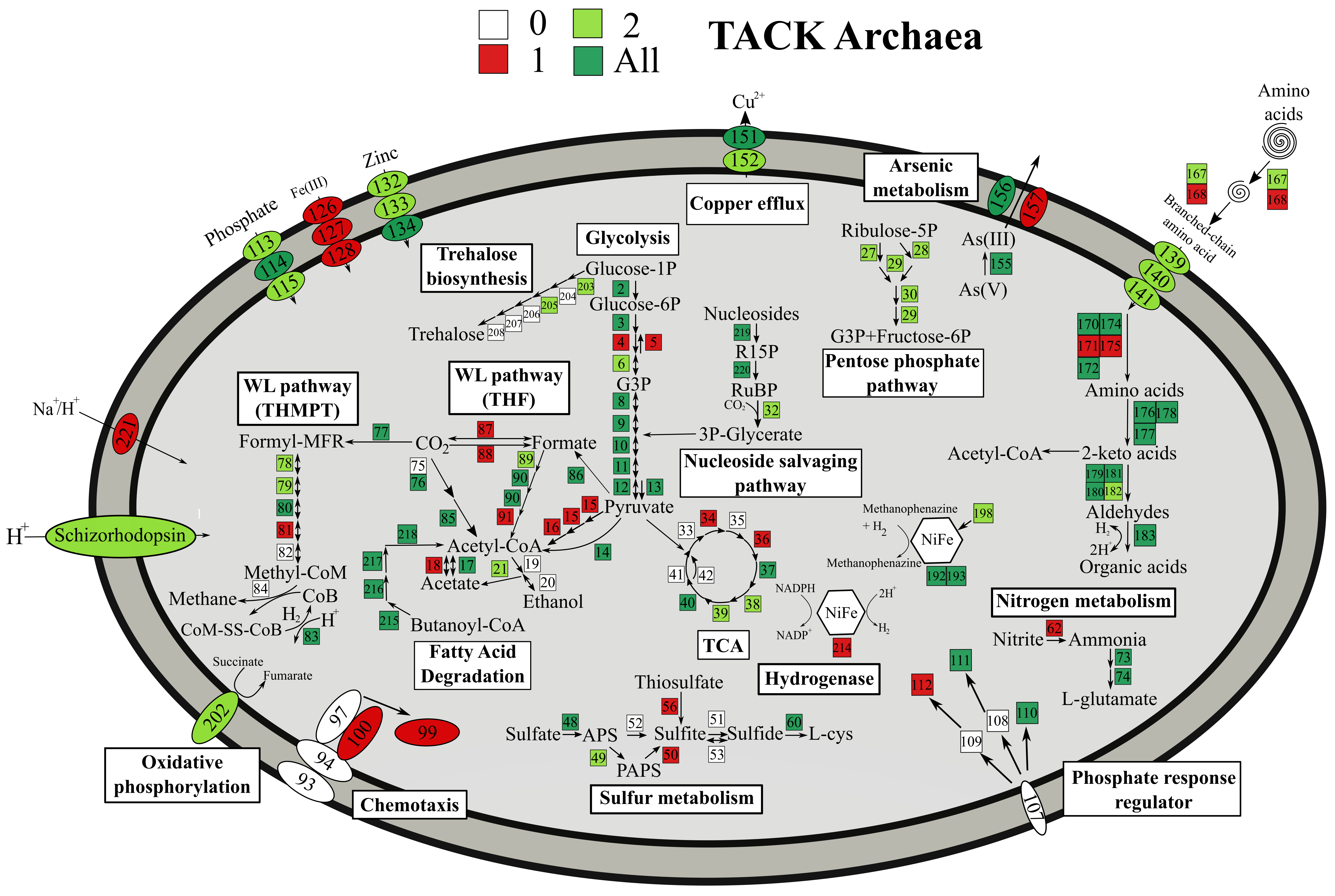

Supplement: Supplementary file 11 — Additional file 10: Figure S9. Metabolic potential of Bathyarchaeota (TACK archaea). A metabolic map summarising the genomic potential and metabolic capacities of the 3 MAGs affiliated with TACK archaea. Numbers represent specific genes in given pathways and the corresponding genes are listed in Additional file 20: Table S3. Different colors in the square boxes represent different numbers of MAGs encoding the genes, while white square boxes indicate the absence of the genes. TCA, tricarboxylic acid cycle; THF, tetrahydrofolate; THMPT, tetrahydromethanopterin; WL pathway, Wood-Ljungdahl pathway; PAPS, 3’-phosphoadenylyl sulfate; APS, Adenylyl sulfate. [file 40168_2020_910_MOESM10_ESM.tiff]

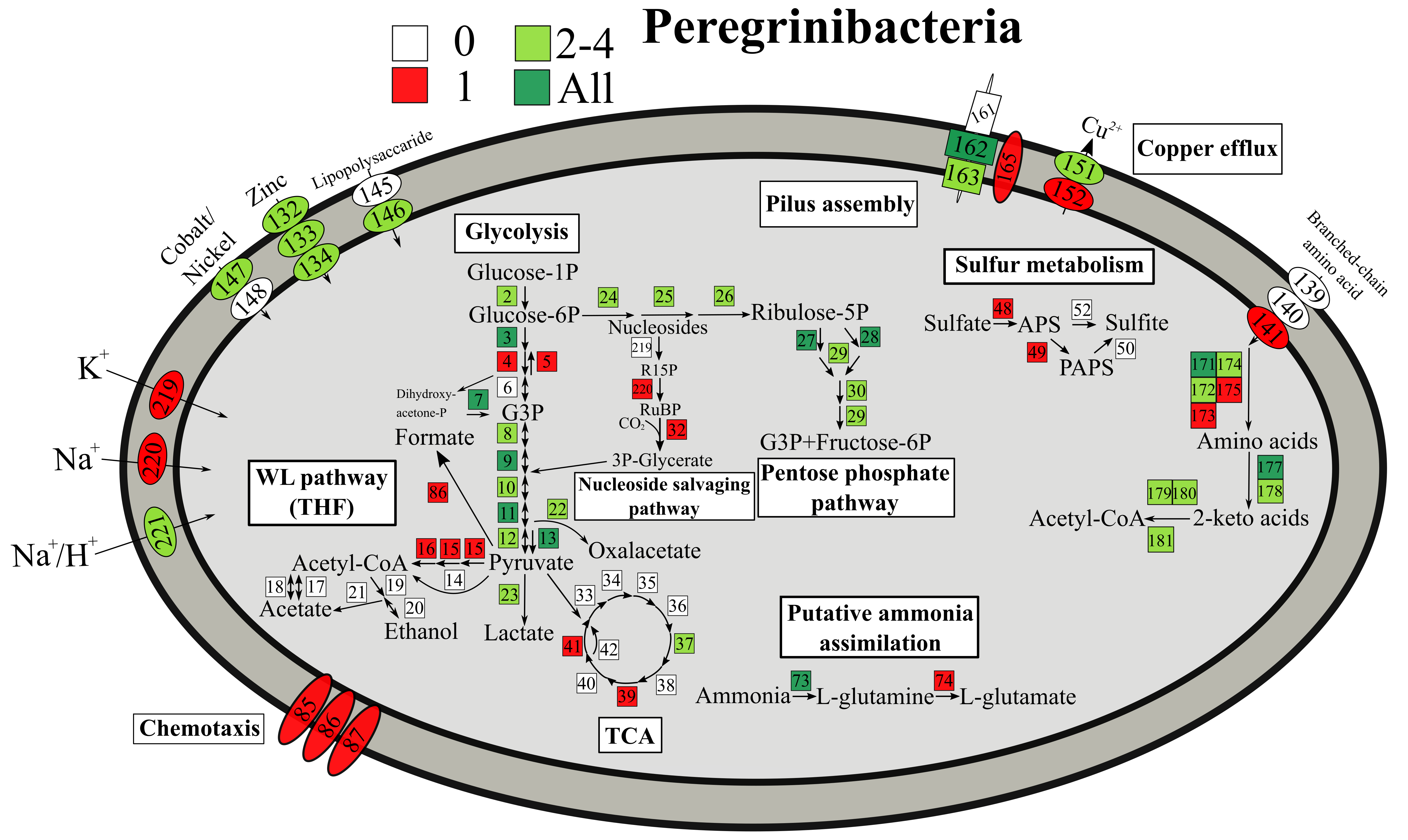

Supplement: Supplementary file 12 — Additional file 11: Figure S10. Metabolic potential of Peregrinibacteria. A metabolic map summarising the genomic potential and metabolic capacities of the 5 MAGs affiliated with Peregrinibacteria. Numbers represent specific genes in given pathways and the corresponding genes are listed in Additional file 20: Table S3. Different colors in the square boxes represent different numbers of MAGs encoding the genes, while white square boxes indicate the absence of the genes. TCA, tricarboxylic acid cycle; THF, tetrahydrofolate; WL pathway, Wood-Ljungdahl pathway; PAPS, 3’-phosphoadenylyl sulfate; APS, Adenylyl sulfate. [file 40168_2020_910_MOESM11_ESM.tiff]

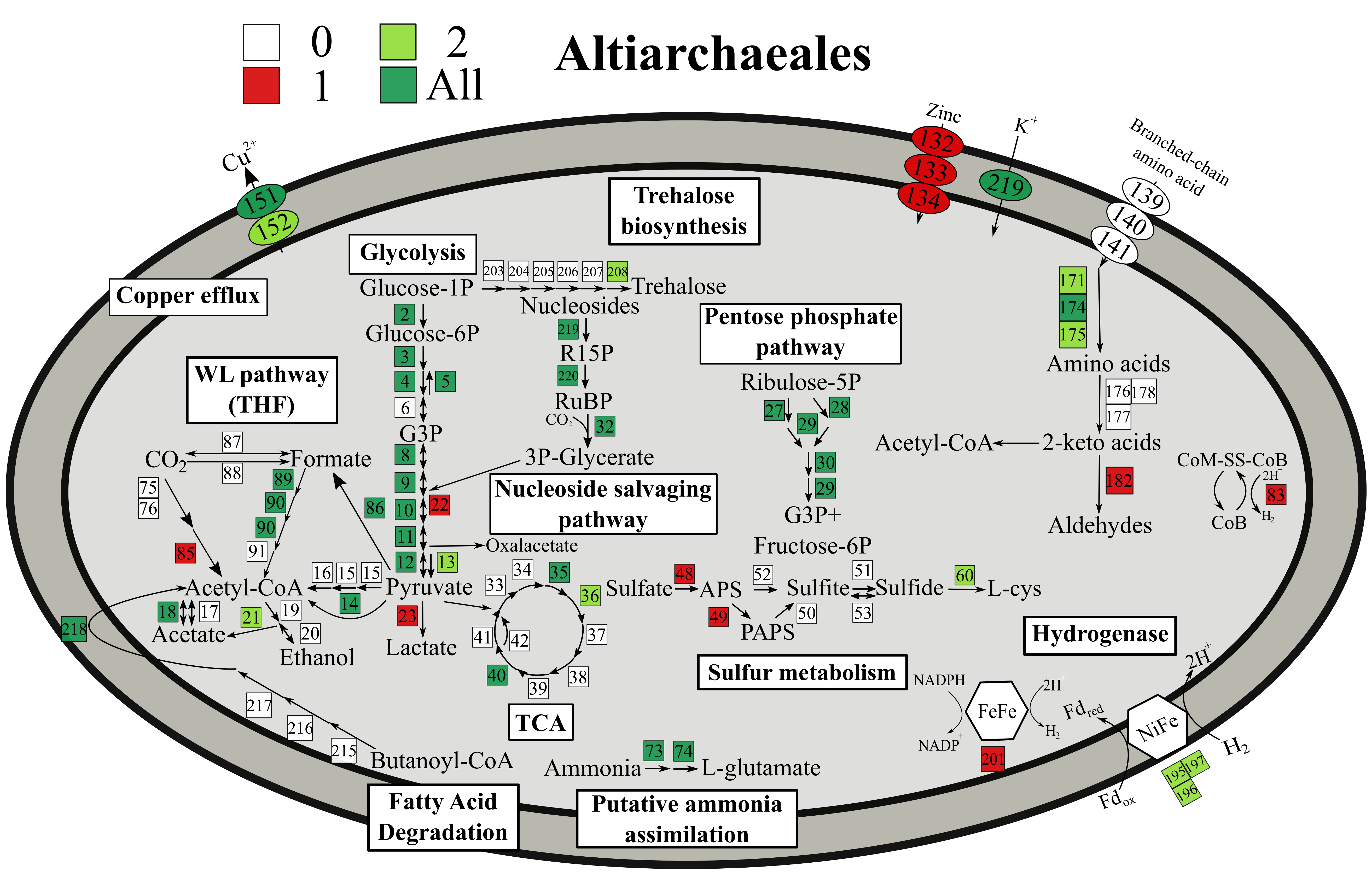

Supplement: Supplementary file 13 — Additional file 12: Figure S11. Metabolic potential of Altiarchaeales. A metabolic map summarising the genomic potential and metabolic capacities of the 3 MAGs affiliated with Altiarchaeales. Numbers represent specific genes in given pathways and the corresponding genes are listed in Additional file 20: Table S3. Different colors in the square boxes represent different numbers of MAGs encoding the genes, while white square boxes indicate the absence of the genes. TCA, tricarboxylic acid cycle; THF, tetrahydrofolate; WL pathway, Wood-Ljungdahl pathway; PAPS, 3’-phosphoadenylyl sulfate; APS, Adenylyl sulfate. [file 40168_2020_910_MOESM12_ESM.tiff]

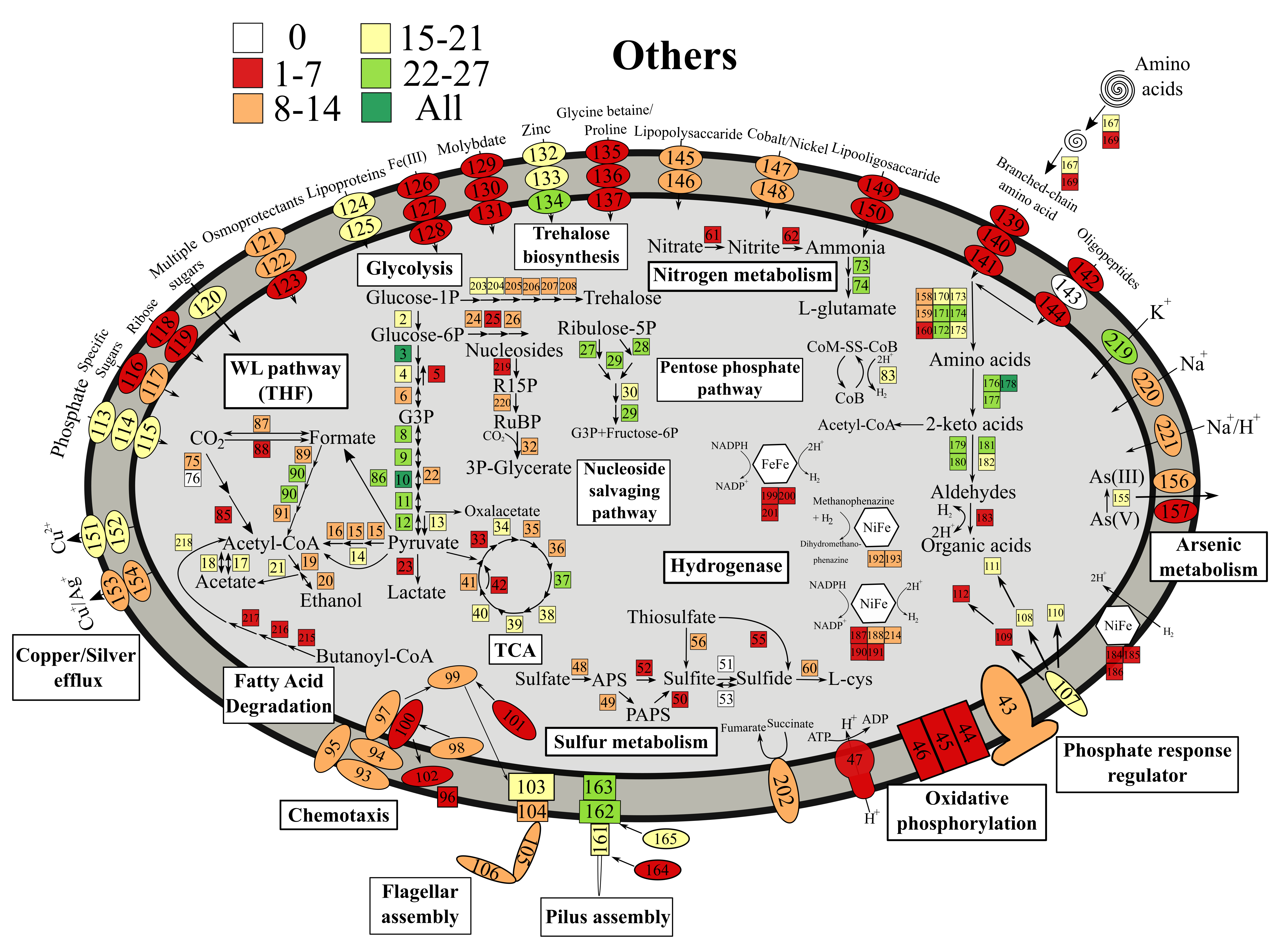

Supplement: Supplementary file 14 — Additional file 13: Figure S12. Metabolic potential of other MDM bacteria. A metabolic map summarising the genomic potential and metabolic capacities of the 28 MAGs affiliated with other MDM bacteria. Numbers represent specific genes in given pathways and the corresponding genes are listed in Additional file 20: Table S3. Different colors in the square boxes represent different numbers of MAGs encoding the genes, while white square boxes indicate the absence of the genes. TCA, tricarboxylic acid cycle; THF, tetrahydrofolate; WL pathway, Wood-Ljungdahl pathway; PAPS, 3’-phosphoadenylyl sulfate; APS, Adenylyl sulfate. [file 40168_2020_910_MOESM13_ESM.tiff]

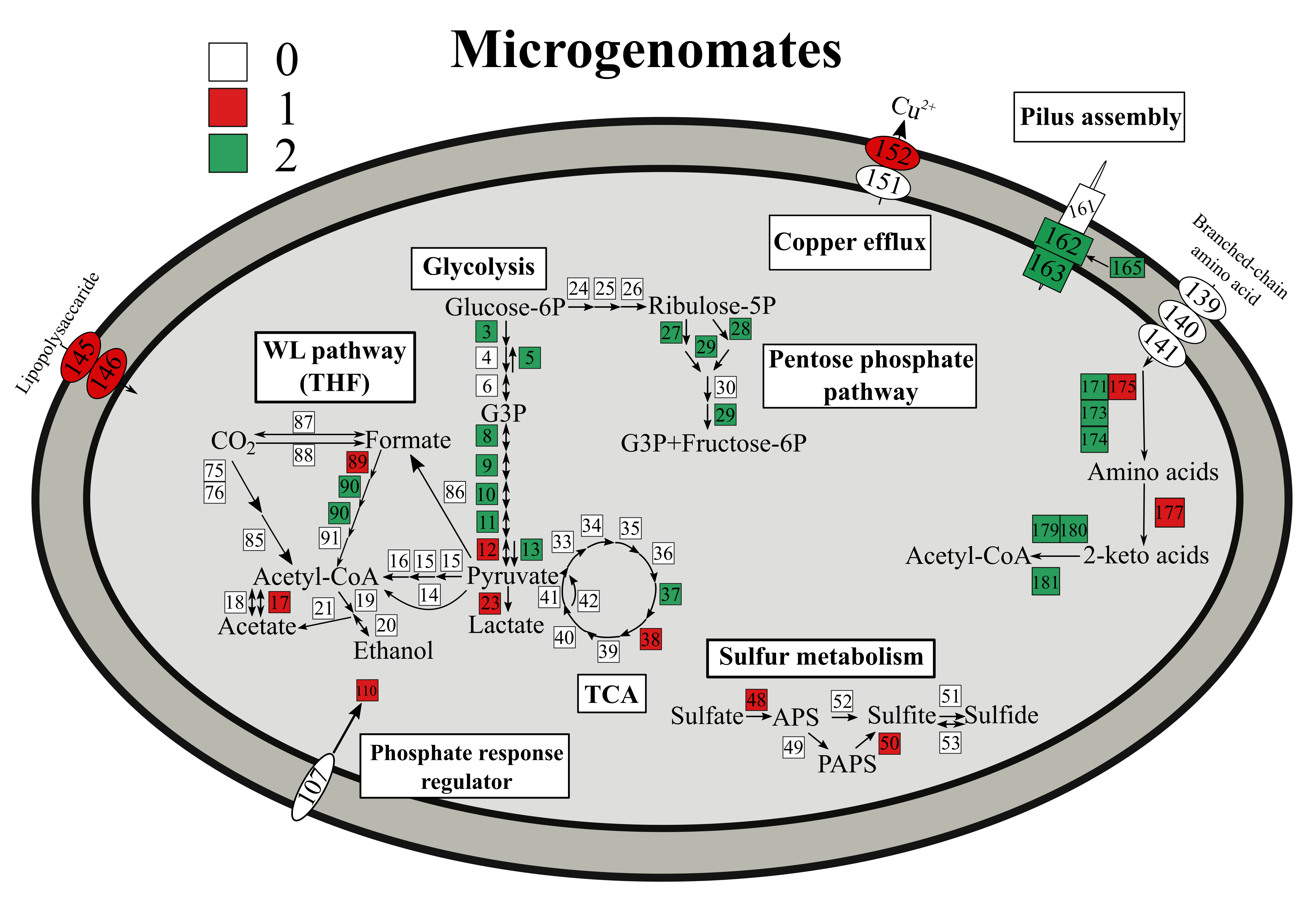

Supplement: Supplementary file 15 — Additional file 14: Figure S13. Metabolic potential of the Microgenomates. A metabolic map summarising the genomic potential and metabolic capacities of the 2 MAGs affiliated with Microgenomates. Numbers represent specific genes in given pathways and the corresponding genes are listed in Additional file 20: Table S3. Different colors in the square boxes represent different numbers of MAGs encoding the genes, while white square boxes indicate the absence of the genes. TCA, tricarboxylic acid cycle; THF, tetrahydrofolate; WL pathway, Wood-Ljungdahl pathway; PAPS, 3’-phosphoadenylyl sulfate; APS, Adenylyl sulfate. [file 40168_2020_910_MOESM14_ESM.tiff]

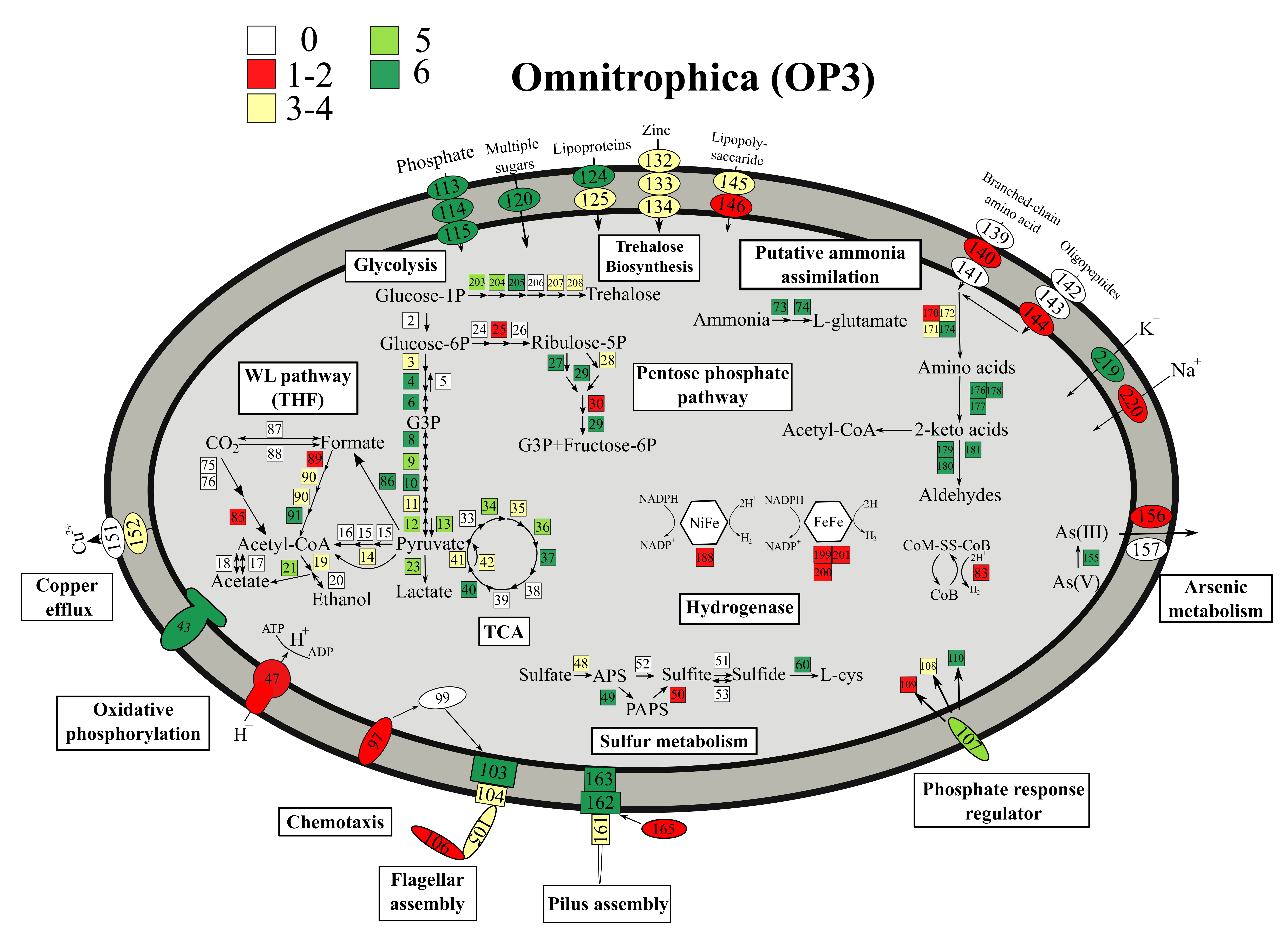

Supplement: Supplementary file 16 — Additional file 15: Figure S14. Metabolic potential of the PVC (Planctomycetes-Verrucomicrobia-Chlamydiae) group bacteria. A metabolic map summarising the genomic potential and metabolic capacities of the 6 MAGs affiliated with Omnitrophica (OP3). Numbers represent specific genes in given pathways and the corresponding genes are listed in Additional file 20: Table S3. Different colors in the square boxes represent different numbers of MAGs encoding the genes, while white square boxes indicate the absence of the genes. TCA, tricarboxylic acid cycle; THF, tetrahydrofolate; WL pathway, Wood-Ljungdahl pathway; PAPS, 3’-phosphoadenylyl sulfate; APS, Adenylyl sulfate. [file 40168_2020_910_MOESM15_ESM.tiff]

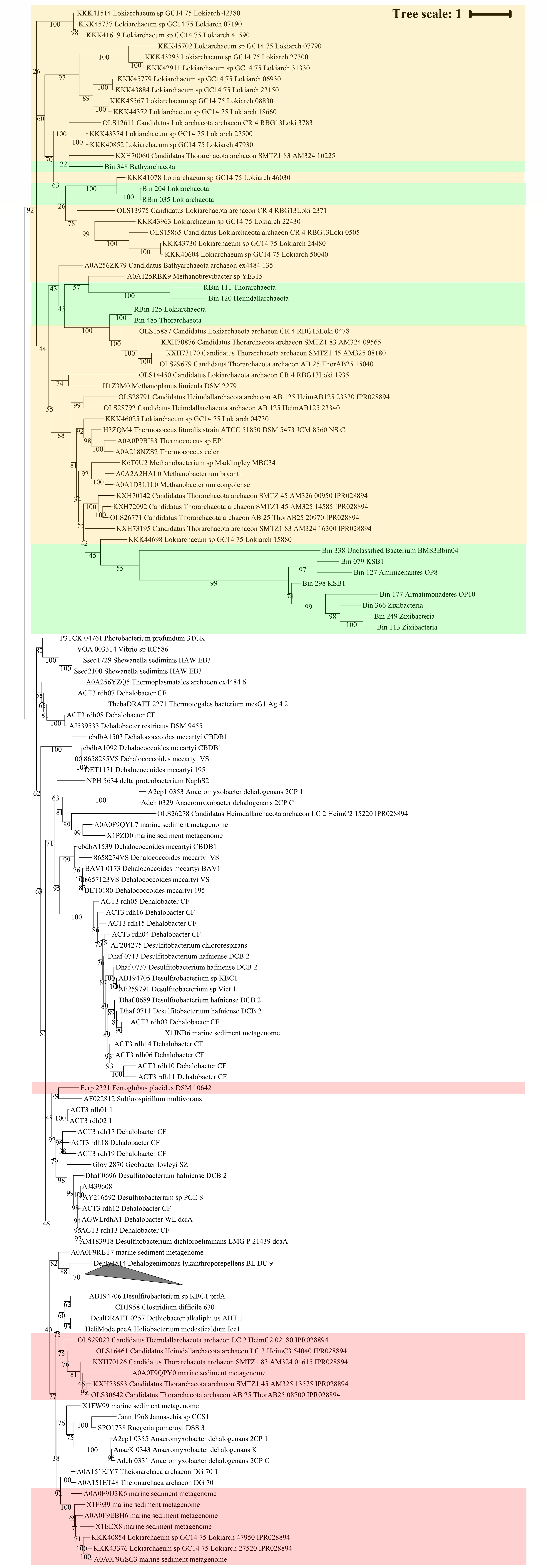

Supplement: Supplementary file 17 — Additional file 16: Figure S15. Maximum-likelihood phylogenetic tree of putative dehalogenase in Shark Bay MDM MAGs. Maximum-likelihood phylogenetic tree constructed with reductive dehalogenase domain (IPR028894) found in the MDM MAGs with 1000 bootstrap replications. Both reductive dehalogenase domain (IPR028894) and epoxyquiuosine reductase were found in Asgard archaea, KSB1, Aminicenantes (OP8), Armatimonadetes (OP10), Zixibacteria and Bathyarchaeota. Although these MAGs encode both epoxyquiuosine reductase and reductive dehalogenase domain, they cluster with homologous sequences of dehalogenase reductases. Thus it is unclear if the MDM community in Shark Bay can respire organohalides. Red shading indicates bona fide dehalogenases found in previous studies [6, 22], yellow shading indicates homologous sequences of dehalogenase reductases, and green shading represent reductive dehalogenase domains (IPR028894) in this study. [file 40168_2020_910_MOESM16_ESM.tiff]

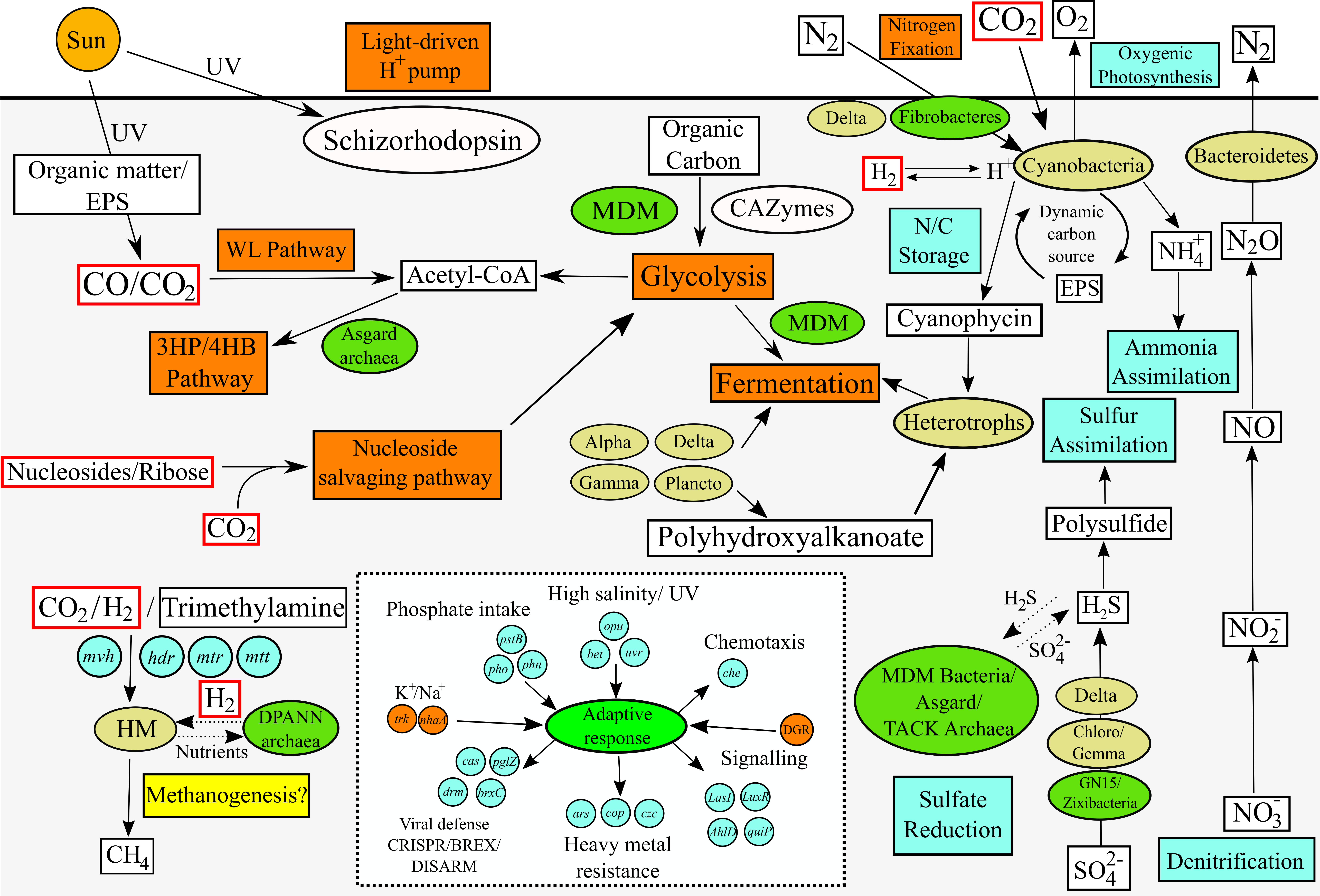

Supplement: Supplementary file 18 — Additional file 17: Figure S16. Proposed ecological model of Shark Bay mats (modified from Wong et al., 2018 [24], incorporating MDM). This is a more detailed ecological model figure incorporating previous findings with putative roles and interactions of MDM. Orange rectangular boxes indicate putative metabolic pathways and putative functional roles of MDM, blue rectangular boxes indicate pathways found in a previous study (Wong et al., 2018 [24]) and white rectangular boxes represent substrates/metabolites. Dark green ovals represent microbial dark matter while light green ovals represent other microorganisms. Red boxes encircle CO2/CO, H2 and ribose that are proposed as main energy currencies of Shark Bay MDM. Dashed arrows indicate putative metabolic exchange/microbial interactions. Dashed box includes genes involved in environmental adaptation. Abbreviation: Chloro, Chloroflexi; Gemma, Gemmatimonadetes; HM, hydrogenotrophic methanogens; N/C storage, nitrogen/carbon storage; WL pathway, Wood-Ljungdahl pathway; 3HP/4HB pathway, 3-hydroxypropionate/4-hydroxybutyrate pathway; EPS, extracellular polymeric substance; DGR, diversity-generating retroelements. [file 40168_2020_910_MOESM17_ESM.tiff]
